# *Ferriphaselus amnicola* strain GF-20, a new iron- and thiosulfate-oxidizing bacterium isolated from a hard rock aquifer

**DOI:** 10.1093/femsec/fiae047

**Published:** 2024-04-04

**Authors:** Mélissa Garry, Julien Farasin, Laetitia Drevillon, Achim Quaiser, Camille Bouchez, Tanguy Le Borgne, Sarah Coffinet, Alexis Dufresne

**Affiliations:** Géosciences Rennes, CNRS, Univ Rennes, UMR 6118, Rennes, France; OSUR, Univ Rennes, UMS 3343, Rennes, France; OSUR, Univ Rennes, UMS 3343, Rennes, France; Ecobio—Ecosystèmes, Biodiversité, Evolution, CNRS, Univ Rennes, UMR 6553, Rennes, France; Ecobio—Ecosystèmes, Biodiversité, Evolution, CNRS, Univ Rennes, UMR 6553, Rennes, France; Géosciences Rennes, CNRS, Univ Rennes, UMR 6118, Rennes, France; Géosciences Rennes, CNRS, Univ Rennes, UMR 6118, Rennes, France; Ecobio—Ecosystèmes, Biodiversité, Evolution, CNRS, Univ Rennes, UMR 6553, Rennes, France; Ecobio—Ecosystèmes, Biodiversité, Evolution, CNRS, Univ Rennes, UMR 6553, Rennes, France

**Keywords:** chemolithoautotrophic, continental subsurface, *Ferriphaselus*, iron oxidation, low-oxygen, thiosulfate-oxidation

## Abstract

*Ferriphaselus amnicola* GF-20 is the first Fe-oxidizing bacterium isolated from the continental subsurface. It was isolated from groundwater circulating at 20 m depth in the fractured-rock catchment observatory of Guidel-Ploemeur (France). Strain GF-20 is a neutrophilic, iron- and thiosulfate-oxidizer and grows autotrophically. The strain shows a preference for low oxygen concentrations, which suggests an adaptation to the limiting oxygen conditions of the subsurface. It produces extracellular stalks and dreads when grown with Fe(II) but does not secrete any structure when grown with thiosulfate. Phylogenetic analyses and genome comparisons revealed that strain GF-20 is affiliated with the species *F. amnicola* and is strikingly similar to *F. amnicola* strain OYT1, which was isolated from a groundwater seep in Japan. Based on the phenotypic and phylogenetic characteristics, we propose that GF-20 represents a new strain within the species *F. amnicola*.

## Introduction

Hard rock aquifers are heterogeneous and dynamic environments hosting a broad diversity of microorganisms (Ben Maamar et al. 2015). In these subsurface environments, primary producers are lithoautotrophs, capable of using energy released by redox reactions to fix CO_2_ and produce organic matter. Microbial diversity studies have highlighted the significant presence of iron-oxidizing bacteria (FeOB) belonging to the family *Gallionellaceae* in geochemically distinct subsurface environments (Ben Maamar et al. 2015, Jewell et al. 2016, Trias et al. 2017, Bethencourt et al. 2020, Bochet et al. 2020). These bacteria oxidize dissolved Fe(II) to obtain energy for their autotrophic metabolism (Hallbeck and Pedersen 1991, Weiss et al. 2007, Emerson et al. 2013, Kato et al. 2014, 2015, Kadnikov et al. 2016, Khalifa et al. 2018). Beside genes involved in iron oxidation and CO_2_ fixation, pathways for sulfur oxidation and nitrate reduction were identified in some genomes of pure cultures and of metagenome-assembled genomes of *Gallionellaceae*, suggesting versatility in their energy metabolism (Emerson et al. 2013, Kato et al. 2015, Jewell et al. 2016, Bethencourt et al. 2020, Hoover et al. 2023). Some *Gallionellaceae* are also characterized by the ability to produce large amounts of rust-colored aggregates, often referred to as “flocs” because of their fluffy appearance. These flocs are composed of twisted stalks corresponding to extracellular structures composed of loosely aggregated Fe-mineralized organic filaments. They have only been described in neutrophilic microaerobic FeOBs, such as the *Gallionellaceae* and *Zetaproteobacteria* (Hallbeck and Pedersen 1990, Chan et al. 2011, 2016: 201, Krepski et al. 2012, Kato et al. 2014).

Metagenomics is a potent approach to obtain a thorough picture of genetic and taxonomic composition of microbial communities. Yet, the need to cultivate microorganisms in order to test hypotheses related to their metabolism, their ecological niches, and their roles in environmental processes remains critical. Very few isolates of *Gallionellaceae* are available in pure culture and all of them originate from iron-rich microbial mats developing in surface environments such as a water drainage system for *Sideroxydans lithotrophicus* ES-1 and *Gallionella ferruginea* ES-2 (Emerson and Moyer 1997), an acid fen peat for *Sideroxydans* strain CL21 (Lüdecke et al. 2010, Cooper et al. 2020), groundwater seeps for *Ferriphaselus globulitus* R-1 (Krepski et al. 2012) and *F. amnicola* OYT1 (Kato et al. 2014), paddy field soil for *Ferrigenium kumadai* An22 (Khalifa et al. 2018), and wetland for *Sideroxyarcus emersonii* MIZ01 (Kato et al. 2022).

*Gallionellaceae* requires microaerobic conditions with high amounts of reduced iron for optimal growth. At circumneutral pH and with high O_2_ concentrations (from 1.5 mg L^−1^, equivalent to 56 µM dissolved O_2_), the chemical oxidation is very fast, with a Fe(II) half-life in the order of a minute, which prevents the growth of neutrophilic FeOB (Emerson et al. 2010, Melton et al. 2014). The biological oxidation predominates at low O_2_ concentrations ranging from ∼0.5 to 50 µM (Neubauer et al. 2002, Druschel et al. 2008, Krepski et al. 2013, Chiu et al. 2017, Maisch et al. 2019). In hard rock aquifers, fractures in the bedrock form networks of flow paths connected by multiple intersections, which allow the mixing of surface oxygenated water with deep anoxic, iron-rich fluids. This provides favorable conditions for FeOB over a large range of depths (Bochet et al. 2020) but the relationship between O_2_ concentration and depth is complex (Osorio‐Leon et al. 2023). O_2_ concentrations fluctuate between 0 and 100 µM, notably because O_2_ delivery to the subsurface is intermittent following seasonal fluctuations of rain events during groundwater recharge periods (Chorover et al. 2017). Consequently, fracture-induced redox gradients are likely temporary and the O_2_ concentration may be the limiting factor controlling the dynamics of iron oxidizer hotspots in the subsurface. Assessing the effect of O_2_ concentration on the growth and metabolism of FeOB is essential to better understand the biogeochemical functioning of the hard rock aquifer.

An additional challenge lies in reproducing the microaerobic conditions encountered by FeOB in experimental setups, especially in the case of low O_2_ concentrations. Most isolation assays and Fe(II) oxidation kinetics studies with FeOBs were performed in glass test tubes with opposing O_2_/Fe gradients (Emerson and Moyer 1997, Druschel et al. 2008, Lueder et al. 2018) or in miniaturized microcosms with liquid cultures and preadjusted O_2_ concentrations in the headspace (Maisch et al. 2019). However, these systems remain limited in their ability to maintain steady conditions and to accurately measure O_2_ concentration throughout incubations, in particular in the case of suboxic range (<5 µM O_2_).

In this work, we isolated the first FeOB representative of hotspots in fractured aquifers. We carried out its physiological and genomic characterization and determined its ecological niche in the continental subsurface. We placed particular emphasis on controlling the oxygen concentration in incubation setups to characterize the physiology of GF-20 at low oxygen concentrations, representative of the suboxic conditions of reduced groundwater with long residence times.

## Materials and methods

### Groundwater sampling and hydrochemical analysis

Groundwater was sampled on 10 January 2022 in an artesian borehole. This borehole is 130 m deep and is located in the discharge area of a fractured rock aquifer near the city of Guidel (Brittany, Western France, PZ26:47°45′13.298″N, 3°28′53.83″W). The aquifer is located in the highly instrumented Ploemeur-Guidel aquifer observatory [French network of hydrogeological observatories H^+^ (http://hplus.ore.fr/en) and French network of Critical Zone observatories OZCAR (http://www.ozcar-ri.org/)]. Many fractures in the crystalline bedrock have been described at different depths in the borehole (Bochet et al. 2020). The deepest fractures supply the borehole with an old (residence time of at least 100 years) anoxic, iron-rich fluid that constitutes the majority of the groundwater upflow (Bochet et al. 2020). The other fractures provide smaller groundwater fluxes, which can contain low concentrations of O_2_ during recharge periods (Bochet et al. 2020).

An inflatable packer was installed at 25 m depth to block the main upflow of deep reducing fluid and to sample only groundwater flowing out of the fracture F20 located at 20 m depth. The isolation of the F20 fracture from the main flux of deep groundwater was confirmed by the suppression of the water flow at the outlet piezometer. Groundwater samples were collected with an MP1 pump (Grundfos). Conductivity, temperature, and pH were measured on-site with a Multi 3620 IDS probe (WTW; with accuracies of ± 0.01 µS cm^−1^, ± 0.1°C, and ± 0.005 pH units). Glass vials of 250 ml were filled and sealed with a rubber septum to avoid exchanges with the atmosphere. For chemical analysis, ∼50 ml of groundwater were filtered at 0.22 µm and stored into polytetrafluoroethylene (PTFE) acid-rinsed bottles. Chemical element concentrations were measured using ICP-MS (HP 4500) and ion chromatography (Dionex DX-120), respectively. Dissolved gases (O_2_, CO_2_, CH_4_, and H_2_) were measured after headspace extraction with a µGC-TCD and chlorofluorocarbons (CFCs) were analyzed using a purge-and-trap GC-ECD.

### Isolation and culture conditions

*Ferriphaselus amnicola* GF-20 was isolated on Modified Wolfe’S Mineral Medium (MWMM: 1 g L^−1^ NH_4_Cl, 0.2 g L^−1^ MgSO_4_.7H_2_O, 0.1 g L^−1^ CaCl_2_.2H_2_O, and 0.05 g L^−1^ K_2_HPO_4_). FeCl_2_ (∼500 µM) was added as a source of Fe(II) (Emerson and Merrill Floyd 2005) in 20 ml of MWMM in 50 ml serum vials sealed with a butyl rubber septum. A headspace gas mixture prepared with a gas mixer (Gas Mixture 100 Series, MCQ Instruments) contained the following composition: O_2_ = 0.25% (corresponding to a concentration of 3 µM O_2_), CO_2_ = 15%, and Ar = 84.75%. The mixture was injected in the vials with a flow rate of 100 ml min^−1^. The pH of the medium was adjusted to 6.8–7.0 with 5 mM NaHCO_3_. For enrichment, 1 ml of groundwater was added to the liquid culture medium. Serial dilutions of 10^−1^−10^−6^ were incubated at 20°C in the dark with agitation. After 72 h, FeOB growth was observable with the presence of typical orange-colored flocs. For isolation, an additional series of dilutions to extinction (10^−9^) were performed. The presence of heterotrophic bacterial contaminants was also tested with growth assays on R2A and LB agar plates incubated at 20°C for 2 weeks.

All precultures for the experiments described below were prepared in MWMM containing 500 µM FeCl_2_, 5 mM NaHCO_3_, and an O_2_/CO_2_/Ar gas mixture (0.25/15/84.75).

### DNA extraction and sequencing

Total DNA was extracted from 3 × 20 ml of pure culture. The culture was centrifuged at 3200×g for 20 min at 4°C in 2 × 50 ml tubes, combined in a 1.5 ml tube, centrifuged at 10 000×g, and the supernatant eliminated. The cell pellet was homogenized in 600 µL of lysis buffer (5% CTAB, 0.7 M NaCl, 240 mM potassium phosphate buffer, pH 7.5, and 2% β-mercaptoethanol) in the presence of glass beads. A volume of 600 µL of phenol–chloroform–isoamyl (PCI, 25:24:1 v/v/v, pH 4.5) was added, vortexed for 1 min, and incubated at 65°C for 5 min. The sample was centrifuged at 11 000×g for 10 min at 4°C. The aqueous phase containing the DNA was transferred to a 1.5-ml tube, 400 µL of isoamyl chloroform (24:1 v/v) was added, vortexed, and centrifuged at 11 000×g for 5 min at 4°C. The aqueous was precipitated with 18% polyethylene glycol overnight, centrifuged at 15 000×g for 30 min at 4°C, purified, and eluted in Tris low ethylene diamine tetraacetic acid (EDTA) buffer (10 mM Tris Ultrapure, 0.1 mM EDTA, and pH 8.0). The DNA amplification was achieved using the GenomiPhi V2 DNA Amplification Kit (Cytiva), following the manufacturer’s instructions. The sequencing library was prepared according to the MGI Easy FS DNA library preparation kit and the sample was sequenced with 2 × 200 bp using a DNBSEQ-G400 high-throughput sequencer (MGI Technology).

### Genome assembly and annotation

The MGI sequencing produced 77 951 062 paired reads. Adapters were removed and sequences with quality scores <25 and <50 bp were trimmed using Cutadapt v2.3 (Martin 2011). Read redundancy was measured with nonpareil v3.3.4 (Rodriguez et al. 2018) and read coverage was normalized with Khmer v3.4.1. To assess the purity of the isolate GF-20, 16S rRNA sequences were reconstructed from metagenomic reads with phyloFlash (Gruber-Vodicka et al. 2020). Reads were assembled with Spades v3.15.5 (Crusoe et al. 2015, Prjibelski et al. 2020). Contigs were assembled using the genome of *F. amnicola* OYT1 as a reference (OYT1_AP018738) with RagTag v2 (Alonge et al. 2022). Completion and contamination were evaluated with CheckM (Parks et al. 2015). The genome sequence of *F. amnicola* GF-20 was annotated using DFAST v1.2.18 with Barrnap and Prodigal options (Tanizawa et al. 2018). The annotation was done manually using the results of KOfamKOALA, BLASTp against the NCBI non-redundant protein sequences database and FeGenie v2.1 tool (Aramaki et al. 2020, Garber et al. 2020). The data for this study has been deposited in the European Nucleotide Archive (ENA) at European Molecular Biology Laboratory-European Bioinformatics Institute (EMBL-EBI) under accession number PRJEB67910 (https://www.ebi.ac.uk/ena/browser/view/PRJEB67910).

### Phylogenetic analyses

DNA sequences were analyzed with phyloFlash (Gruber-Vodicka et al. 2020) to confirm the presence of a unique 16S rRNA gene sequence and the purity of the culture GF-20. The average nucleotide identity (ANI) and the average amino acid identity (AAI) were calculated with the enveomics collection (Rodriguez and Konstantinidis 2016). DNA–DNA hybridization (DDH) was calculated using the Genome-to-Genome Distance Calculator version 3.0 (Meier-Kolthoff et al. 2014). Sequences similar to the GF-20 16S rRNA gene sequence were searched in the SILVA database version 138 with SINA aligner version 1.2.11 (Pruesse et al. 2012). A Maximum Likelihood (ML) phylogenetic tree was inferred from the multiple alignment of 16S rRNA gene sequences using the TN+G+I model with 1000 bootstrap iterations with MEGA v11.0.8 (Tamura et al. 2021: 11). In addition, 120 bacterial marker genes present in single copy were retrieved from the genomes of GF-20 and 9 other isolates of *Proteobacteria* with GTDB-tk v2.1 and the GTDB database release 07-RS207 (Chaumeil et al. 2022, Parks et al. 2022). Amino-acid sequences of the marker genes were concatenated into large sequences (5024 amino acids), which were subsequently aligned with MUSCLE (Edgar 2004). A phylogenomic ML tree was constructed from multiple alignments using the LG+G model and 1000 bootstrap iterations with the MEGA software.

### Physiological characteristics

Temperature, salinity, and pH tolerance were determined by the presence of microbial flocs observed after 72 h of incubation. The temperature range of 4, 6, 10, 20, 30, 35, and 40°C and NaCl concentrations of 0, 0.05, 0.1, 0.15, 0.2, 0.3, and 0.5% (w/v) were tested. The pH range tested was 5.4, 5.8, 6, 7, 8, and 8.5. For pH 5.8, 0.5 mM NaHCO_3_ was added in MWMM medium. For pH 6, 2.5 mM NaHCO_3_ and for pH 7, 5 mM NaHCO_3_ were added in MWMM medium. For pH 8 and 8.5, 15 and 20 mM NaOH were added accordingly. All conditions were tested with 3 µM O_2_ concentration and 500 µM FeCl_2_.

### Microscopic observations

The culture was observed with scanning electron microscopy (SEM, JEOL IT 300) to identify microbial characteristic structures. Several samples of microbial flocs were fixed with 50% EtOH and deposited on a nylon filter membrane (diameter = 25 mm and pore size = 0.2 µm, Whatman) or glass slide to dehydrate, then dried out at the critical point (Balzers Instruments, CPD010). The samples were metalized with gold/palladium with the Leica EM ACE200 before the observations with the SEM.

### Fatty acid analysis

Fatty acids were extracted, purified, and methylated as described by Elvert et al. (2003). In brief, biomass of 150 ml of pure culture was harvested by multiple centrifuge steps (10 000×g at 4°C for 20 min) and freeze-dried. The freeze-dried residue was saponified with a 15% methanolic NaOH solution for 3 h at 80°C. The neutral lipid fraction was separated by liquid–liquid extraction with cyclohexane. The pH was then set to 1 by dropwise HCl addition and the fatty acid fraction was purified by liquid–liquid extraction with cyclohexane. Methylation of the fatty acid fraction was performed with 0.5 ml of a 20% BF_3_ solution in methanol for 1 h at 70°C. Fatty acid methyl esters (FAMEs) were identified by gas chromatography–mass spectrometry (QP2010+MS, Shimadzu) using the standard FAME Mix (47885-U, Supelco) and the NIST Mass Spectral Library (NIST 05). The integration of FAMEs was performed on the major ion (Supplementary Table S3) and the quantification was performed by the addition of perdeuterated n-alkanes as internal standards (eicosane, tetracosane, triacontane, CDN, and Sigma). The GC-MS was operated in electron impact mode at 70 eV with a full scan mass range of 50–600 m/z. The chromatographic separation was performed on a fused silica SLB-5MS capillary column (length = 60 m, diameter = 0.25 mm, and film thickness = 0.25 µm, Supelco) under the following oven temperature program: 70°C (held for 1 min) to 150°C at 15°C min^−1^, then 150–300°C (held for 15 min) at 3°C min^−1^. The chromatograph was coupled to the mass spectrometer by a transfer line heated to 280°C, and he was fed with a constant flow rate (flow = 1 ml min^−1^) as carrier gas.

### Electrons donors and acceptors

To determine the ability of GF-20 to grow with substrates other than Fe(II), triplicate cultures were prepared with different electron acceptors and donors under suboxic and anoxic conditions (Table 3). The anoxic condition was obtained by adding cysteine (5 mM) and resazurin (4 µM) to the culture media and with a gas mixture composed of CO_2_ (15%) and Ar (85%) (Wagner et al. 2019). Growth of GF-20 with four inorganic substrates: FeSO_4_ (500–600 µM), NH_4_Cl (1 mM), Na_2_S_2_O_3_ (1 mM), and MnSO_4_ (5 mM) were tested in suboxic conditions (O_2_ = 0.25%). Similarly, five organic molecules: glucose, sucrose, pyruvate, lactate, and acetate (5 mM each) were used as potential electron donors to evaluate the heterotrophic growth of GF-20 under suboxic conditions. Growth under anoxic conditions was tested with three inorganic acceptors: NaNO_3_ (1 mM), Na_2_S_2_O_3_ (1 mM), and MnSO_4_ (5 mM) and glucose (5 mM) as electron donors. Fermentation was also assessed with glucose, sucrose, pyruvate, lactate, or acetate (5 mM). For every tested substrate, cultures were inoculated with 1 ml of preculture containing 10^5^ cells mL^−1^, pH was adjusted to 6.8–7 by adding NaHCO_3_ (5 mM). The headspace was renewed every day during the experiments. Growth was evaluated by the presence of FeOB flocs or by random view field counting of cells stained with Syto13 (5 µM, Invitrogen™) on an epifluorescence microscope (DM-IL LED, Leica) after 72 h. A negative control without inoculation was carried out for each condition.

To investigate the effect of thiosulfate oxidation coupled with Fe(II) oxidation, different conditions were set up under suboxic conditions. These included a condition with FeCl_2_ (600 µM) and NaS_2_O_3_ (1 mM), a condition with only NaS_2_O_3_ (1 mM), and a condition with only FeCl_2_ (600 µM). Each condition was performed in triplicate using a mineral medium (MWMM) culture, and a negative control without bacteria was included. Other negative control conditions with only the MWMM medium were also prepared. For each culture, the pH was adjusted to 6.8–7 by adding NaHCO_3_ (5 mM), inoculated with 1 ml of preculture containing 10^6^ cells mL^−1^. All conditions were tested in suboxic conditions (O_2_ = 0.25%, CO_2_:15%, and Ar:84.75%) and the headspace was renewed every day. Ferrous iron and thiosulfate concentrations were determined with spectrophotometric methods (UV_mini_ 1240, Shimadzu, accuracy ± 60 µM) with 1,10-phenantroline (270 mM) and acetate ammonium (5 M) in 50% of acetic acid (Harvey et al. 1955) and with Na-acetate trihydrate (6.25 mM) and acetic acid (6.25 mM) (Dupraz et al. 2019) pH with a pH microelectrode (SI Analytics, accuracy ± 0.3). Both were measured twice a day during 72 h. Stained cells with Syto13 (5 µM, Invitrogen™) were counted in random view fields using epifluorescence microscope (DM-IL LED, Leica) equipped camera (C13440, Hamamatsu) to determine the cell abundance twice a day during 72 h. The ferrous iron and thiosulfate consumptions as a function of the number of cells were calculated.

### Oxygen dependence of Fe(II) oxidation rate

To determine the optimal O_2_ concentration for the growth of GF-20, seven concentrations were tested: 0, 1, 3, 13, 26, 44, and 58 µM. For each concentration, a gas mixture was adjusted with CO_2_ (15%) and O_2_/Ar (v/v) and used as headspace. A volume of 1 ml of preculture containing 10^5^ cells mL^−1^ was used to inoculate 50 ml of MWMM supplemented with 500–600 µM FeCl_2_ and buffered to pH 6.8–7.0 with 5 mM NaHCO_3_ (Emerson and Merrill Floyd 2005). To subtract the abiotic oxidation of Fe(II) from the total Fe(II) oxidation, negative controls without bacteria were made. All conditions were performed in triplicate and incubated under dark and agitation at 20°C. Fe(II) concentrations were determined with 1,10-phenanthroline spectrophotometric method (UVmini 1240, Shimadzu, accuracy ± 60 µM) (Harvey et al. 1955), pH with a pH microelectrode (SI Analytics, accuracy ± 0.3), and O_2_ concentrations with a non-invasive optical oxygen sensor (SP-PSt8-YAU, Presens, detection limits: 0.1–140 µM, accuracy ± 0.02 µM). These three parameters were measured every 12 h during a 72-h period. To maintain the O_2_ concentration, the headspace was renewed daily. Abiotic and biotic Fe(II) oxidation rates were calculated between 21 and 50 h (described in the Supplementary Data) and the contribution of the process was calculated as in Maisch et al. (2019).

## Results

### Field description and physicochemical parameters of groundwater

Groundwater collected in the F20 fracture had a circumneutral pH (pH 6.8), a high conductivity (506 µS cm^−1^), and a temperature of 14.9°C. The chemical composition was classical of a silicate bedrock aquifer (Supplementary Table S1), with particularly high concentrations of iron (0.04 mM), manganese (0.02 mM), and sulfate (0.4 mM^1^). Low O_2_ (0.90 µM) and CFCs (<0.15 pM) concentrations were measured, indicating reduced water with a residence time of at least 70 years (Ayraud et al. 2008).

### Isolation of twisted stalk iron-oxidizing bacteria

Strain *F. amnicola* GF-20 was isolated on MWMM medium by applying three successive series of dilutions to extinction. Rust-orange microbial flocs were visible after 24 h of incubation at 20°C, indicating the development of FeOB (e.g. images of microbial flocs in Supplementary Fig. S2). We obtained a pure culture since only one 16S rRNA gene sequence was reconstructed from the high-throughput sequencing of the genomic DNA extracted from the final culture. In addition, no bacterial colonies grew on the R2A and LB agar plates and microscopic observations indicated a homogeneous culture (results not shown).

**Figure 1.**
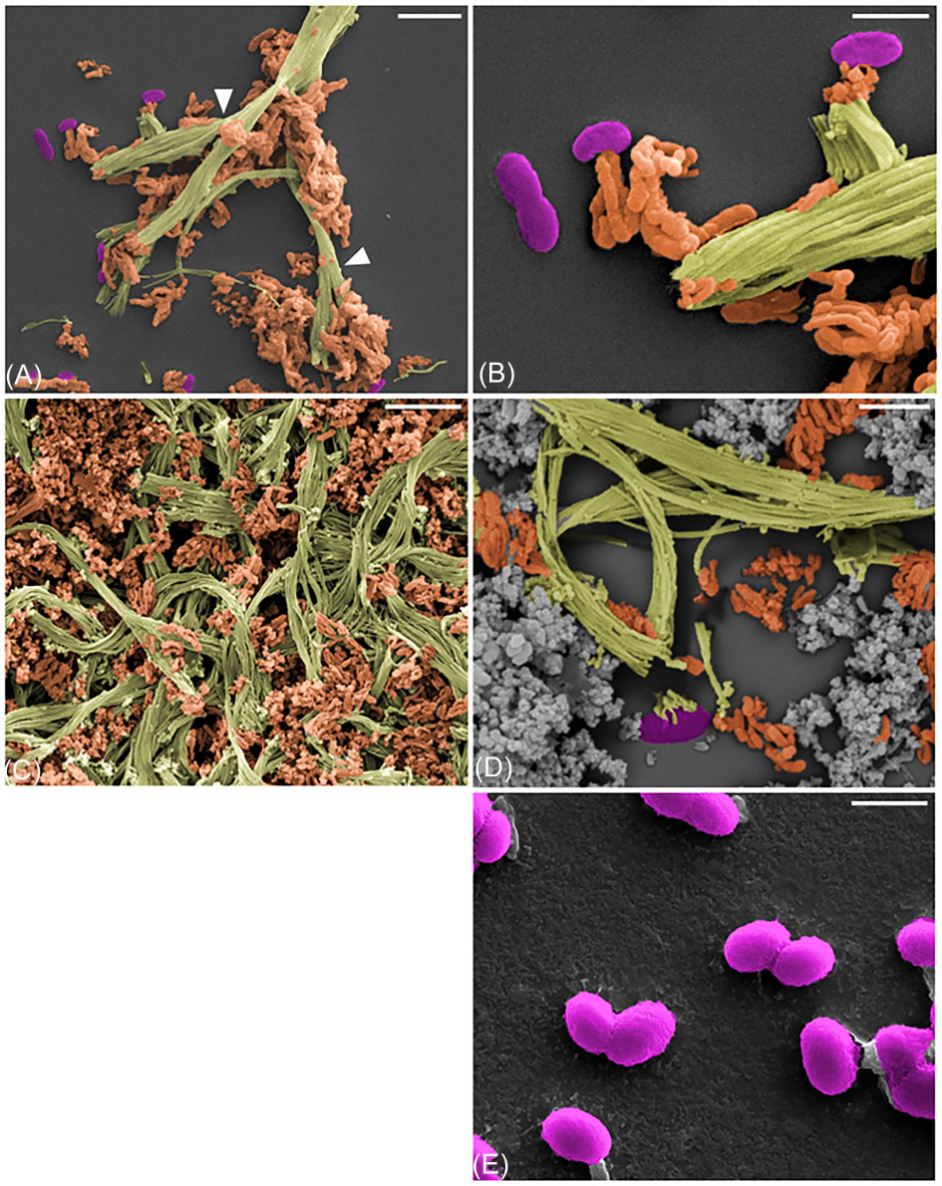
Colorized SEM images of GF-20 cultures. (A and B) Culture of GF-20 with only FeCl_2_, scale bars: 5 and 2.5 µm, respectively. (C and D) Culture of GF-20 with FeCl_2_ and thiosulfate, scale bars: 5 and 2.5 µm, respectively. (E) Culture of GF-20 with only thiosulfate, scale bar: 1 µm. Dread extracellular structures are shown in orange and twisted stalk structures in green. GF-20 cells are colored purple and Fe oxides produced by abiotic oxidation are shown in gray. White arrows indicate branching in the extracellular stalks that reflect cell division events.

Microscopic observations showed that the cells of GF-20 were curved rods of 0.8–1.5 µm in length with a diameter of 0.4–0.6 µm (Fig. 1). They were motile (results not shown) and secreted typical twisted stalks (Fig. 1). Other types of extracellular structures, similar to the amorphous clusters of Fe oxide minerals “dreads” described in *F. amnicola* OYT1 (Kato et al. 2015, McAllister et al. 2019) were also visible.

### Taxonomic affiliation and genomics features of *F. amnicola* GF-20

**Figure 2.**
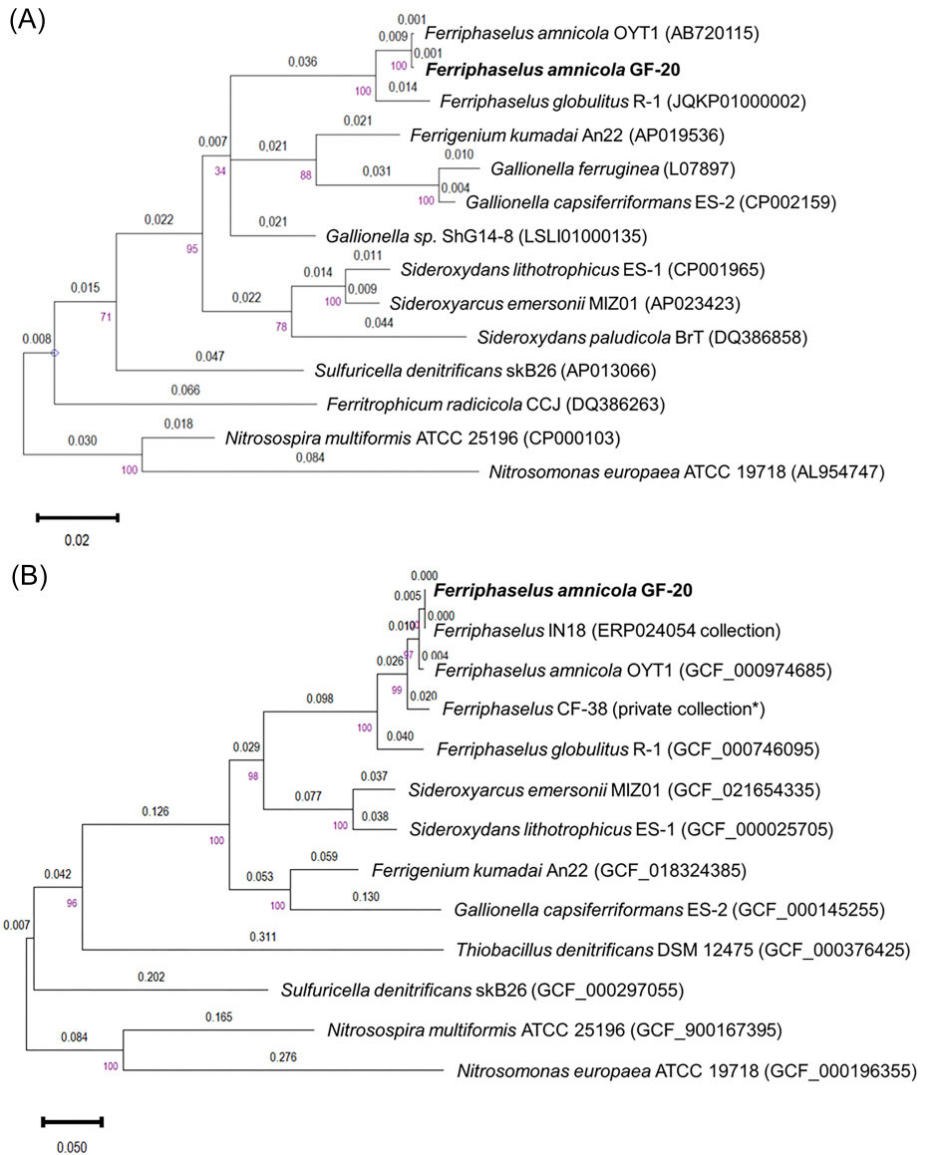
Phylogeny of strain GF-20. (A) 16S rRNA tree; (B) phylogenomic tree based on amino acids of 120 single-copy genes. For both trees, distances are shown on the branches and bootstrap values (1000 replicates) are shown on the nodes. *Nitrosospira multiformis* ATCC 25196 and *Nitrosomonas europaea* ATCC 19718 were used as an outgroup for both trees. *MAG CF-38 reconstructed from samples (Trias et al. 2017) and used in the pangenome analysis (Hoover et al 2023).

Genomic DNA was extracted from GF-20 cultures to reconstruct the genome sequence and to validate the purity of the isolate. Three 16S rRNA gene copies were found in the assembled genome sequence of GF-20 and were identical to a unique 16S rRNA gene sequence reconstructed from the metagenomic reads with phyloFlash. The 16S rRNA gene showed 99.9% identity to the 16S rRNA gene of *F. amnicola* OYT1, which was above the commonly used species threshold of 98.7% (Table 1) (Rosselló-Móra and Amann 2015). Phylogenetic analyses based on the 16S rRNA gene showed that strain GF-20 formed a single clade with strains *F. amnicola* OYT1 and *F. globulitus* R-1 (Fig. 2) (Kato et al. 2015). This clade was well separated from the other genera defined in the family *Gallionellaceae*, such as *Gallionella, Sideroxydans*, and *Sideroxyarcus*. Phylogenomic analysis based on single-copy genes also indicated that GF-20 was extremely close to the metagenome-assembled genome (MAG) *Ferriphaselus* IN18 (Fig. 2B). This MAG was also reconstructed from PZ26 groundwater metagenomes (Bethencourt et al. 2020).

**Table 1.** Genomic characteristics of strain GF-20 and related strains of the genus *Ferriphaselus*.

Strains	*F. amnicola* GF-20	*F. amnicola* OYT1^T^	*F. globulitus* R-1	MAG *Ferriphaselus* CF-38	MAG *Ferriphaselus* IN18
**Genomic characteristics**					
Accession number	PRJEB67910	AP018738	ASM74609v1	Private collection	ERP024054
Completeness (%)	98.00	98.34	98.34	80.94	87.97
Contamination (%)	0.20	0.59	0.00	6.54	7.55
Number of contigs	10	1	23	195	79
Size (bp)	27 51 500	2 7 17 229	24 41 319	22 15 221	28 08 320
G+C content (%)	55.5	55.9	60.7	56.7	55.4
CDS	2654	2614	2371	2036	2673
Number of tRNA genes	48	49	50	31	41
Number of rRNA genes	9	9	12	0	1
**Similarity indices against F-20**					
16S rRNA		99.93%	97.87%	nd	nd
ANI		96.10%	83.36%	87.98%	99.91%
AAI		97.08%	85.15%	90.90%	99.92%
DDH		65.9–71.7%	21.6–28.6%	29.9–34.8%	97.8–99%
**Genes for iron and sulfur oxidation**					
*cyc2*	+	+	+	nd	+
*mtoAB*	nd	−	nd	nd	nd
*sox*	nd	−	nd	nd	nd
*dsr*	+	+	+	nd	+

nd: genes not found because the genome is incomplete.

The ANI and AAI values between GF-20 and IN18 were both 99.9% (Table 1). The ANI and AAI values between GF-20 and OYT1 were 96 and 97%, respectively (Table 1). These values were above the threshold for species circumscription (Konstantinidis and Tiedje 2005, Rosselló-Móra and Amann 2015). Altogether, these results indicated that GF-20 belongs to the species *F. amnicola* but constitutes a different strain.

The genome of GF-20 was assembled into 1 scaffold of 10 contigs. The genome measured 2 751 500 bp long and encoded 2639 protein-coding genes. Completeness and contamination were estimated at 98% and 0.2%, respectively, with CheckM. Genomic features are summarized in Table 1 and Supplementary Table S2. Genome annotation revealed the presence of genes involved in the oxidation of ferrous iron and sulfur, microaerobic respiration, and fixation of carbon. For iron oxidation, the GF-20 genome had only one copy of the cluster 1 *cyc2* gene (Garber et al. 2020, Keffer et al. 2021). Genes of the porin-cytochrome c protein complex (PCC) (*mtoAB, mtrD*, and *pioAB*), as well as the genes encoding for the cytochromes CymA and Cyc1 were not found (Supplementary Table S2) (He et al. 2017). A complete set of *act* genes coding for the alternative complex III (*actAB1B2CDEF*) was present.

For dissimilatory sulfur oxidation, the GF-20 genome encoded a reduced set of genes, including the dissimilatory sulfite reductase (*dsr ABEFHCMKLJOPN*), the sulfite reductase (*soeABC*), and the sulfide quinone oxidoreductase (*sqr*) genes. Genes coding for the proteins of the Sox complex (*soxABXYZ*), the sulfate adenylyltransferase (sat), the adenosine 5′-phosphosulfate reductase (*aprABM*), the acetyltransferase (*ttr*), the sulfotransferase (ssu), and the sulfate-binding protein (*sbp*) were missing in the GF-20 genome.

For electron transport, genes encoding NADH dehydrogenase (Complex I), succinate dehydrogenase (Sdh) (Complex II), cytochrome-*bc1* ubiquinol oxidase (Complex III), and Cbb_3_-type cytochrome *c* oxidase (Complex IV) were detected. For energy conservation, F-type ATP synthase (Complex V) coding genes were found in the GF-20 genome. For carbon fixation, the GF-20 genome had genes for the Calvin–Benson–Bassham (CBB) cycle, including ribulose-1,5 bisphosphate carboxylase/oxygenase (RuBisCO Form II, *cbbM*). All genes for the Embden–Meyerhof–Parnas and pentose phosphate pathways and the tricarboxylic acid cycle (TCA) were present in the GF-20 genome. For nitrogen fixation, a large set of *nif* genes (*nifHDKTWZBQXNE)* was present.

A cluster of genes potentially involved in stalk formation in OYT1 (Kato et al. 2015), including a *bcsB* (cellulose synthase regulator) and the *xagBCD* genes (extracellular polysaccharide production), was found in the GF-20 genome. The latter also contained the *bcsABZC-like* genes, which were hypothesized to be involved in cellulose synthesis for the production of dreads in OYT1 (Kato et al. 2015). For the attachment, the motility and the chemotaxis genes coding for the Type IV pilus system (*pilAFGHJMNOPQRS*), the flagellum complex system (*fliAEFGHIJMNQRS, flgABCDEFGHIJKL*, and *flhABCD*), the chemotaxis proteins (*cheABDRWYZ*), and the aerotaxis receptor (Aer) were found in the GF-20 genome. Superoxide dismutase B (SodB) was found as a defense system against oxidative stress but the gene encoding the enzyme catalase (Cat) was not identified.

### Fatty acid profile

The fatty acid profile of GF-20 contained: C10:0 (0.48%), C12:0 (1.31%), C14:0 (3.44%), C15:1 (0.37%), C15:0 (3.18%), C16:1 (0.43%), C16:0 (42.94%), C17:0 (2.00%), C18:2ω6t and/or C18:3ω3 and/or C18:1ω9c (0.90%), C18:0 (24.42%), C20:0 (2.32%), C22:1 (0.46%), C22:0 (2.32%), C23:0 (3.14%), C24:0 (6.34%), and C26:0 (5.94%) (Supplementary Table S3). Comparison of the fatty acid profiles of *F. amnicola* GF-20 and other related isolated FeOBs (Table 2) indicated that C16:0 was common as the major fatty acid. The second major fatty acid was C18:0 for *F. amnicola* GF-20, like *F. globulitus* R-1 strains (Kato et al. 2015).

**Table 2.** Morphological and physiological characteristics of strain GF-20 and of related strains of *Gallionellaceae*.

Strains	1	2	3	4	5	6	7	8	9
Cell morphology	Curved rod	Curved rod	Curved rod	Bean-shaped	Curved or helical rod	Curved or helical rod	Curved rod	Curved rod	Curved rod
Cell diameter (µm)	0.4–0.6	nd	0.7–0.9	0.5–0.8	0.32	0.3–0.5	0.42	0.2–0.4	0.73
Cell length (µm)	0.8–1.5	1.8–2.1	0.8–1.9	0.8–2.5	nd	1.0–2.4	nd	0.9–2.0	nd
Division time (h)	10.5	15	10.9	8.3	8	12.4–19.8	15.8	6.2	12.5
Stalk-forming	+	+	+	+	−	−	−	−	−
Temperature range for growth (°C)*	6–30 (15–20)	10–35 (25–30)	8–30 (25–30)	5–25 (20)	10–35 (30)	10–40 (30–35)	19–37 (nd)	12–37 (25–30)	6–21 (nd)
pH range for growth*	6–8 (6.4–7)	5.6–7.0 (5.6–6.1)	5.6–7.3 (6.1–6.5)	5.5–6.5 (nd)	5.5–7.0 (6.0–6.5)	5.5–7.0 (6.0)	4.5–7.0 (nd)	5.2–6.8 (5.9–6.1)	5.5–7.0 (6.0–6.5)
NaCl tolerance (%)	<0.3	<0.3	<0.8	nd	nd	<0.2	nd	<0.15	nd
Thiosulfate oxidation	+	−	−	+	+	+	−	−	−
Major fatty acids^∆^	16:0 18:0	16:0 18:0	16:0 16:1*w*7c/ 16:1*w*6c	nd	16:0 16:1*w*7*c*/iso15:02-OH	16:0 16:1*w*7c/ 16:1*w*6c	16:0 16:1*w*7c/ 16:1*w*6c	16:0 16:1*w*7c/ 16:1*w*6c	16:0 18:0 18:1*w*9c 16:1*w*7c/iso 15:02-OH

Strains: (1) *F. amnicola* GF-20 (this study); (2) *F. globulitus* R-1 (Krepski et al. 2012, Kato et al. 2015); (3) *F. amnicola* OYT1T (Kato et al. 2014); (4) *G. ferruginea* (Hallbeck and Pedersen 1990, Lütters-Czekalla 1990, Hallbeck et al. 1993); (5) *S. lithotrophicus* ES-1 (Emerson and Moyer 1997, Emerson et al. 2013); (6) *S. emersonii* MIZ01T (Kato et al. 2022); (7) *S. paludicola* BrT (Weiss et al. 2007); (8) *F. kumadai* An22T (Khalifa et al. 2018); and (9) *G. capsiferriformans* ES-2 (Emerson and Moyer 1997, Emerson et al. 2013). nd: not determined.

* Optimum values in parentheses.

∆ Fatty acids with relative abundance ≥10%.

### Conditions and substrates for the growth of *F. amnicola* GF-20

GF-20 grew between 6–30°C (optimally at 15–20°C) and pH 6–8 (optimally at 6.4–7) (Table 2, Supplementary Fig. S2). The strain tolerated 2 mg L^−1^ (35 mM) NaCl, but no growth was observed at 3 mg L^−1^ (50 mM) NaCl (Table 2, Supplementary Fig. S1c).

Incubations with different electron donors and acceptors were carried out in anoxic (0 µM O_2_) and suboxic (3 µM O_2_) conditions to assess the metabolic capacities of GF-20. Under the suboxic condition, GF-20 grew chemolithoautotrophically, oxidizing iron and thiosulfate. No growth was observed with the other tested organic and inorganic substrates in the presence of O_2_ (Table 3, Supplementary Fig. S3). In anoxic condition, GF-20 did not grow regardless of the tested substrate (Table 3, Supplementary Fig. S3).

**Table 3.** Growth tests of *F. amnicola* GF-20 on mineral and organic substrates.

Electrons acceptors	Growth	Electron donors	Growth
In suboxic condition:			
O_2_	+	FeCl_2_	+
		FeSO_4_	+
		NH_4_Cl	−
		Na_2_S_2_O_3_^a^	+
		MnSO_4_^b^	–
		Glucose	−
		Sucrose	−
		Pyruvate	−
		Lactate	−
		Acetate	−
In anoxic condition:			
NaNO_3_	−	Glucose	−
MnSO_4_^b^	−		
Na_2_S_2_O_3_^a^	−		
Glucose^c^	−		
Sucrose^c^	−		
Pyruvate^c^	−		
Lactate^c^	−		
Acetate^c^	−		

a Na_2_S_2_O_3_ can be used as an electron donor coupled to O_2_ or as an electron acceptor coupled to a reduced substrate such as glucose.

b MnSO_4_ was used to test the reduction of sulfate in anoxic condition and the oxidation of manganese in microoxic condition.

c In anoxic condition, organic substrates were used as both electron acceptors and electron donors to test fermentation.

**Figure 3.**
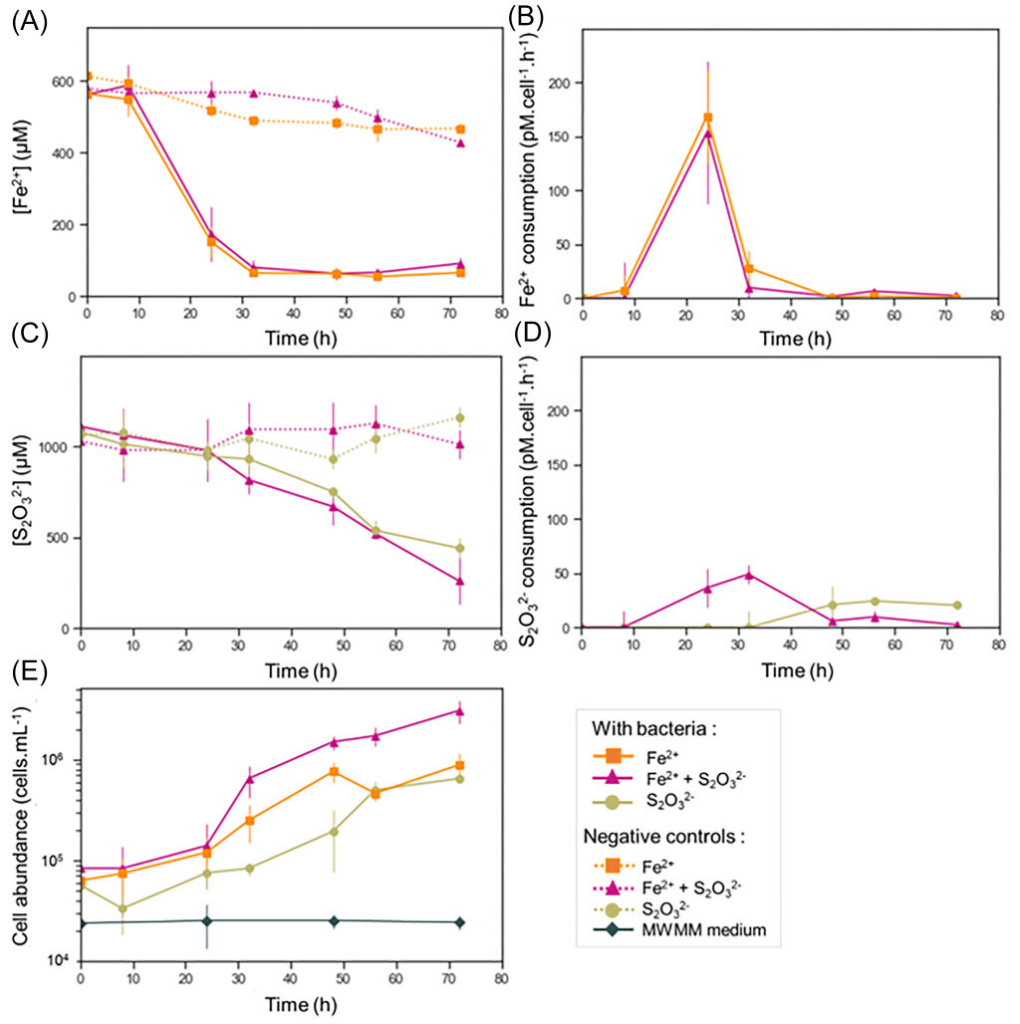
Concentration and consumption of Fe(II) and thiosulfate, and cell abundance in cultures of GF-20. (A and C) Fe(II) and thiosulfate concentrations in GF-20 cultures incubated with FeCl_2_ only, thiosulfate only or FeCl_2_ and thiosulfate. (B and D) Iron and thiosulfate consumption in GF-20 cultures with FeCl_2_ only, thiosulfate only or FeCl_2_ and thiosulfate. (E) Cell abundance in GF-20 cultures. Negative controls (dotted lines), which corresponds to incubations without bacteria with the exception of the MWMM medium condition (gray diamond) that correspond to incubations with bacteria but without any addition of electron donor. Values represented in the plots are the averages of the measurements made on the triplicates. Error bars correspond to the standard deviations.

Incubations with FeCl_2_ and FeSO_4_ were characterized by the development of orange flocs in the culture medium. GF-20 was also able to grow with NaS_2_O_3_ as the sole electron donor as observed by increasing cell numbers (Fig. 3E, Supplementary  Fig. S4). Under these conditions, flocs were not produced, indicating that the growth was not associated with the production of twisted stalks (Fig. 1E).

To gain insights into the Fe and thiosulfate oxidative metabolisms, incubations were carried out in MWMM medium with iron (FeCl_2_) and thiosulfate (Na_2_S_2_O_3_) and compared to incubations with iron or thiosulfate alone. For the incubations with FeCl_2_ only and FeCl_2_ + Na_2_S_2_O_3_, microbial flocs were visible after 24 h (Fig. 3, Supplementary Fig. S4). Iron was oxidized at nearly identical rates in both conditions (Fig. 3A–B). The concentration of iron decreased rapidly and reached a plateau after 32 h of incubation, suggesting that iron became limiting. On the contrary, the oxidation of thiosulfate pursued up to the end of the incubation but it appeared slower than that of Fe (Fig. 3C–D). This was partly due to a longer lag time for the oxidation of thiosulfate compared to Fe (Fig. 3A–C). The lag time also seemed to last longer in the case of incubation with Na_2_S_2_O_3_ alone than with the combined addition of Na_2_S_2_O_3_ and FeCl_2_. Rate of thiosulfate oxidation normalized to the cell abundance peaked higher and earlier when thiosulfate was associated with iron than with thiosulfate alone (Fig. 3D). The effect of the nature of the electron donor could also be seen in the growth of GF-20. It was faster with FeCl_2_+ Na_2_S_2_O_3_ than with iron alone, while it was faster with iron than with thiosulfate alone (Fig. 3E).

### Effects of O_2_ concentrations on the development of *F. amnicola* GF-20 and Fe(II) oxidation rates

To determine the influence of O_2_ concentration on the development of GF-20 and the oxidation of Fe, incubations with O_2_ concentrations ranging from 0 to 58 µM were performed. O_2_ concentrations were kept stable during the incubations and iron concentrations were monitored (Supplementary Fig. S5). The growth of GF-20 was confirmed by the presence of typical microbial flocs of FeOB in the culture medium.

**Figure 4.**
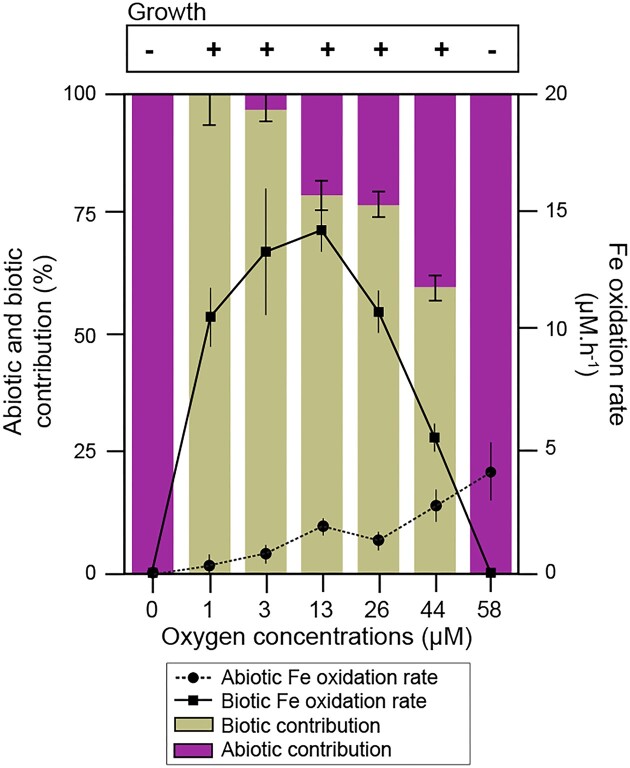
Relative contribution of abiotic and biotic oxidation to total Fe(II) oxidation and abiotic and biotic Fe oxidation rates in GF-20 cultures as function of oxygen concentration. Rates and contributions are average values measured in triplicates. Error bars correspond to standard deviation.

In anoxic condition (0 µM O_2_), the Fe(II) concentration did not change during the experiments (Supplementary Fig. S5). No microbial floc or iron oxide precipitates were observed and the abiotic and biotic Fe(II) oxidation rates were both negligible, with values of 0.29–0.35 µM h^−1^, respectively (Fig. 4, Supplementary Fig. S2d, Supplementary Table S4). Orange flocs were visible in the culture medium from 1 to 44 µM O_2_ (Supplementary  Fig. S2d). A strong increase of the biotic oxidation rate was observed at 1 µM O_2_ compared to the anoxic condition (Fig. 4). Between 1 and 26 µM O_2_, the biotic oxidation rate was always >10 µM h^−1^. This rate peaked at an intermediate O_2_ concentration (13 µM) and was ∼7–30 times higher than the abiotic rate under these conditions. Therefore, the contribution of biotic oxidation to the total Fe(II) oxidation ranged from 100% at 1 µM to 78% at 26 µM O_2_. Flocs could still be observed with an O_2_ concentration of 44 µM but the biotic rate was only twice the abiotic rate and their relative contribution was more balanced (Fig. 4). Finally, the abiotic oxidation rate was maximum (4.2 µM h^−1^) at 58 µM O_2_. Neither floc production nor biotic oxidation were detected at this O_2_ concentration but a thin orange deposit probably corresponding to iron oxides was visible (Fig. 4, Supplementary  Table S4).

## Discussion

### *Ferriphaselus amnicola* GF-20, is representative of the continental subsurface

*Ferriphaselus amnicola* GF-20 was successfully isolated from groundwater collected at 20 m depth in the PZ26 borehole of the Guidel aquifer observatory in Brittany. However, groundwater flows were ascending in the borehole (PZ26) and temperature indicated a water depth origin of several hundred meters (Osorio‐Leon et al. 2023), which is consistent with measured long residence time.

GF-20 was highly similar to the MAG IN18, which was described in a previous metagenomic characterization of borehole PZ26 (Bethencourt et al. 2020). In this study, a large diversity of *Gallionellaceae* was observed in the groundwater flowing through the fractures and in the iron-rich microbial mats outside the surface casing of the borehole. A clear separation in the distribution of the *Gallionellaceae* lineages was detected between deep groundwater and the microbial mat on the surface. In particular, *Gallionellaceae* MAGs, which dominated the surface microbial mats, were not found in the groundwater metagenomes. This strongly suggests that *Gallionellaceae* developing on the surface cannot colonize the deep fractured layer of the aquifer (Bethencourt et al. 2020). Consequently, *F. amnicola* GF-20 is likely representative of reduced continental subsurface environments and groundwaters with long residence time. To our knowledge, this is the first strain of *Gallionellaceae* isolated from groundwater directly sampled in the continental subsurface. The conditions measured in PZ26 are found in the other boreholes of the discharge zone of the catchment (Osorio‐Leon et al. 2023), as well as in other deep silicate environments (Bochet et al. 2020). Therefore, this strain is potentially present in many deep surface environments.

### High genomic similarity between geographically isolated *F. amnicola* strains

The ANI and AAI values, 96–97%, respectively, indicated that GF-20 and OYT1 belong to the same species but to different strains. OYT1 was isolated from an iron-rich microbial mat at a groundwater seep in Japan (Kato et al. 2014). The strong similarity at the genomic level was unexpected since the two strains came from different environments separated by a very significant geographical distance (∼10 000 km). Analysis of the effects of geographic separation on evolutionary relationships among >35 000 microbial genomes suggested a strong geographic endemism and a low dispersal rate for subsurface microorganisms compared to marine and terrestrial microorganisms (Louca 2022). Analysis of the similarity between microbial genomes in relation to their geographical origin revealed that the probability of finding two subsurface-associated strains with an ANI value >95% (i.e. two strains assigned to the same species) on opposite hemispheres of the Earth or even on different continents was almost zero. The high degree of similarity between the genome sequences of GF-20 and OYT1 despite their undeniable geographical isolation raises fundamental questions. It may be explained by a lower rate of genome evolution as was advanced for another subsurface dweller, the bacterium “Candidatus *Desulforudis audaxviator*” (Becraft et al. 2021). However, the mechanism behind this unexpected genome stability must still be explored.

### Versatile metabolism for electron donors

*Ferriphaselus amnicola* GF-20 is a facultative iron oxidizer because it could grow autotrophically using thiosulfate as the sole electron donor. This is a striking difference with OYT1 and R-1, which could not be cultivated on thiosulfate (Krepski et al. 2012, Kato et al. 2014). The high similarity between GF-20 and OYT1 raises the possibility that the non-detection of thiosulfate oxidation in OYT1 may be related to the experimental conditions rather than to the composition of its protein-coding gene repertoire.

In the family *Gallionellaceae*, the capacity to grow by thiosulfate oxidation alone has only been demonstrated in *S. lithotrophicus* ES-1 (Emerson et al. 2013, Zhou et al. 2022), *G. ferruginea* (Lütters-Czekalla 1990), and *S. emersonii* MIZ01 (Kato et al. 2022). In addition, it was recently shown that ES-1 can simultaneously oxidize iron and thiosulfate as GF-20 (Zhou et al. 2022). Consistently with their physiology, ES-1 and MIZ01 harbored the genetic repertoire necessary for the growth on thiosulfate. An incomplete Sox pathway (SoxXYZAB) was found in ES-1 (Emerson et al. 2013) while the thiosulfate dehydrogenase (TdsAB) was identified in both ES-1 (Zhou et al. 2022) and MIZ01 (Kato et al. 2022). In addition, ES-1 and MIZ01 may also generate sulfate via their reverse-acting dissimilatory sulfite reductase (Dsr) and sulfite-oxidizing enzyme (Soe). In comparison with ES-1 and MIZ01, the three *Ferriphaselus* strains shared genes encoding for Dsr and Soe but not Sox and TsdAB. This limited set of sulfur oxidation genes might be associated with a lower growth on thiosulfate compared to ES-1 and MIZ01. Growth assays with ES-1 and MIZ01 cultures resulted in higher cell densities with thiosulfate alone than with iron (Kato et al. 2022, Zhou et al. 2022); the opposite situation was observed with cultures of GF-20. However, it cannot be excluded that GF-20 uses a still-unknown system to catalyze the first step of thiosulfate oxidation or that these genes were not detected due to an incomplete genome.

### Stalk production linked to iron oxidation

The production of stalks and dreads was observed in GF-20 cultures with ferrous iron alone and ferrous iron and thiosulfate. These structures were not seen in cultures with only thiosulfate. It is worth noting that GF-20 cells cultivated on thiosulfate produced stalks once transferred in fresh medium containing iron. Current hypotheses suggest that extracellular structures observed in FeOB, such as stalks, may help cells prevent encrustation by Fe oxides or control their position in O_2_ gradients (Krepski et al. 2013, Chan et al. 2016). Our findings support the hypothesis of a functional link between the production of stalks and dreads and the oxidation of iron. A candidate gene cluster for stalk production was identified in *Ferriphaselus* strains OYT1 and R-1 and stalk-forming *Zetaproteobacteria* of the genus *Mariprofundus* (Kato et al. 2015, Koeksoy et al. 2021). Another cluster of genes putatively involved in the formation of dreads has been identified specifically in OYT1 and R-1 (Kato et al. 2015). All these genes were also detected in the GF-20 genome. Since O_2_ concentrations were the same in all incubations testing the oxidation of iron and thiosulfate, our results confirmed that the production of extracellular structures depends primarily on the presence of ferrous iron. Metatranscriptomic studies or site-directed mutagenesis (CRISPR) assays, to inactivate candidate genes involved in the formation of stalks or dreads, appear necessary to better understand the relationships between the formation of extracellular structures, iron oxidation and growth.

### Effects of O_2_ concentrations on GF-20 iron oxidation activity

One noteworthy advantage of our culturing method is the possibility to test the effect of low O_2_ concentrations (<5 µM O_2_), which are particularly difficult to maintain (Neubauer et al. 2002, Maisch et al. 2019), on biotic iron oxidation. In GF-20 cultures, the optimum concentration corresponding to the maximum biotic oxidation rate was estimated between 3 and 13 µM O_2_, which is consistent with the concentrations measured in PZ26. Our findings also revealed that the favorable O_2_ concentration range for GF-20 was shifted to very low concentrations, as shown by the abrupt increase of the biotic oxidation rate from 0 to 1 µM O_2_, which accounts for the total iron oxidation at this O_2_ concentration. *Ferriphaselus amnicola* GF-20 is the first isolated *Gallionellaceae* strain able to oxidize iron at O_2_ concentration of <2 M. It shares this capacity with M*ariprofundus aestuarium* CP-5, a marine FeOB isolated from the Chesapeake Bay oxic–anoxic transition zone (Chiu et al. 2017). Furthermore, the biotic contributions to the iron oxidation are much higher than previous estimates for a *Sideroxydans* enrichment from a rice paddy field incubated in a similar setup with liquid medium cultures and preadjusted O_2_ concentrations (Maisch et al. 2019). We postulate that the ability to oxidize iron at very low O_2_ concentrations and with high efficiency may be useful for GF-20 to thrive in reduced fractures of the hard rock aquifers where O_2_ inputs are low and scarce.

## Conclusions

Iron-oxidizing bacteria (FeOB) belonging to the family *Gallionellaceae* are often detected in subsurface environments. However, FeOB are not homogeneously distributed in the subsurface and their abundance is fluctuating with time, as a result of localized and intermittent O_2_ arrival. Understanding the effect of O_2_ concentration on the growth and metabolism of subsurface FeOB is thus key to better characterize the microbial habitability of the subsurface. *Ferriphaselus amnicola* GF-20 is the first FeOB isolated directly from the continental subsurface and representative of an old and reduced groundwater environment. GF-20 is chemolithoautotrophic and can exploit particularly low O_2_ concentrations to oxidize iron and produce stalks. This suggests an important contribution of these bacteria to carbon fixation and primary production in the subsurface. As GF-20 could be also cultivated on thiosulfate, our findings also extend the geochemical niche of *Ferriphaselus amnicola*. This study advocates the need to obtain microbial isolates adapted to the conditions of the subsurface to gain insights into the functioning of underground ecosystems.

### Description of *F. amnicola* strain GF-20

*Ferriphaselus* (Fer.ri.pha.se'lus. L. neut. n. *ferrum* iron; L. masc. n. *phaselus* bean; N. L. masc. n. *Ferrifaselus* iron bean). *Ferriphaselus amnicola* [am.ni'co.la. L. masc. n. *amnis* a stream, a small river; L. suff. - *cola* (from L. n. *incola*) a dweller, an inhabitant; N. L. masc. n. *amnicola* an inhabitant of a stream] (Kato et al. 2014). Cells are gently curved, short rods (0.4–0.8 and 0.8–1.5 mm). Produce an extracellular twisted stalk from the concave side of the cell. Motile. Gram-negative. Do not form spores. Mesophilic and neutrophilic. Subaerobic and microaerobic (1–44 µM O_2_). Autotrophic. Capable of oxidizing Fe(II) and thiosulfate as an energy source for lithotrophic growth. Does not utilize nitrate, sulfate, ammonium, Mn(II), pyruvate, glucose, or sucrose as an energy source and does not grow with acetate and lactate. Grows at 6–3°C (optimally at 15–20°C) and pH 6.0–8.0 (optimally at pH 6.4–7.0). The doubling time under optimal conditions is 10.5 h. Grows at low salt concentrations, <3.0 g NaCl L^−1^. The major cellular fatty acids are C16:0 and C18:0. Phylogenetically close to *F. amnicola* OYT1 (99.93% sequence similarity of the 16S rRNA gene) but with different genetic content (96% ANI and 97% AAI).

## Supplementary Material

fiae047_Supplemental_File
