# Supplementary material for: Ferriphaselus amnicola strain GF-20, a new iron- and thiosulfate-oxidizing bacterium isolated from a hard rock aquifer
Source: FEMS Microbiol Ecol. 2024 Apr 4;100(5):fiae047. doi: 10.1093/femsec/fiae047 (PMC11044966; doi:10.1093/femsec/fiae047)
Supplement: fiae047_Supplemental_File [file fiae047_supplemental_file.docx]

*Ferriphaselus amnicola* strain GF-20, a new iron and thiosulfate-oxidizing bacterium, isolated from a hard rock aquifer.

Authors: Garry, M.^1,2^, Farasin, J.^2^, Drevillon, L^.3^, Quaiser, A^.3^, Bouchez. C.^1^, Le Borgne, T.^1^, Coffinet, S.^3^, Dufresne, A^.3^

Affiliations :

1: Univ Rennes, CNRS, Géosciences Rennes, UMR 6118, Rennes, France

2: Univ Rennes, CNRS, OSUR, UMS 3343, Rennes, France

3: Univ Rennes, CNRS, Ecobio - Ecosystèmes, Biodiversité, Evolution, UMR 6553, Rennes, France

1. **Calculation of Fe(II) oxidation rates**

The total oxidation of Fe(II) (FeOx_tot_) is a combination of abiotic (FeOx_abio_) and biotic (FeOx_bio_) homogeneous oxidation reactions, and a heterogeneous autocatalytic (FeOx_auto_) oxidation reaction. The autocatalytic heterogeneous oxidation of dissolved Fe(II) by Fe(III) mineral products, accelerates the oxidation of Fe(II) (Tamura, Goto and Nagayama 1976).

The oxidation rates of each reaction are calculated as follows :

**(Equation 1)** $\frac{{FeOx}_{auto}}{dt}=\left( \frac{k_{s,0}\times\left[ O_{2} \right]\times K}{\left[ H^{+} \right]} \right) \times\left[ Fe\left( III \right) \right]\times\left[ Fe\left( II \right) \right]_{tx}$

with k_s,0_ = 73 mol.L^-1^.s^-1^, K = 10^-4.85^ , $\left[ Fe\left( III \right) \right] = \left[ Fe\left( II \right) \right]_{t0}-\left[ Fe\left( II \right) \right]_{tx}$ , where tx = 50 hours

**(Equation 2 for abiotic setup)** ${FeOx}_{abio}= {FeOx}_{tot} - {FeOx}_{auto}$

**(Equation 3 for biotic setup)** ${FeOx}_{bio}= {FeOx}_{tot} - ({FeOx}_{abio}+{FeOx}_{auto})$

All oxidation rates were calculated during the incubation phase from 21 to 50 h, *i.e.* after the lag phase, when the initially available Fe(II) started to be oxidized and the differences between the biotic and abiotic setups were the most important.

Based on the abiotic and biotic Fe(II) oxidation rates, the microbial contribution on total Fe(II) oxidation was calculated as described by (Maisch *et al.* 2019) :

**(Equation 4)**

$$Biotic contribution \left( \% \right)= \frac{\left[ Fe\left( II \right) \right]_{tx, abio}- \left[ Fe\left( II \right) \right]_{tx, bio}}{\left[ Fe\left( II \right) \right]_{t0, bio}-\left[ Fe\left( II \right) \right]_{tx, bio}} \times100$$

1. **Subcultures of the culture only thiosulfate (no stalk) in an MWMM medium with Fe.**

To verify that iron is required for the production of stalks, we inoculated 20 mL of MWM medium amended with 500 µM of FeCl_2_ with 1 mL of a GF-20 preculture grown on 1 mM of thiosulfate and containing 6.48x10^5^ cells per mL. The growth was tested in suboxic conditions (O_2_ = 0.25%, CO_2_ : 15%, Ar : 84.75%). The pH was adjusted to 6.8-7 by adding NaHCO_3_ (5 mM). The presence of microbial flocs was observed after 24 h. This confirmed that the production of stalks and the formation of flocs take place when a source of Fe(II) is added to the culture medium and that GF-20 can achieve iron oxidation.

**Table_S1**_**Supplementary_Data**. Concentration of major ions and dissolved gases of the sampled groundwater

| Concentrations (mM) | | | | Gases (µM) | | CFC (pM) | |
| --- | --- | --- | --- | --- | --- | --- | --- |
| Anions | | Cations | |  |  |  |  |
| F^-^ | 0,01 | Na | 1,93 | O_2_ | 0,90 | CFC-12 | 0,12 |
| Cl^-^ | 2,35 | Mg | 1,51 | CO_2_ | 599,28 | CFC-11 | 0,05 |
| SO_4_^2-^ | 0,40 | Si | 0,48 | CH_4_ | 0,79 | CFC-113 | 0,02 |
| Br^-^ | 0,005 | Ca | 0,47 | H_2_ | 0,02 |  |  |
| NO_3_^2-^ | 0,03 | Mn | 0,02 |  |  |  |  |
|  |  | Fe | 0,04 |  |  |  |  |
|  |  | As | 0,004 |  |  |  |  |

**Table_S2_Supplementary_Data.** Comparison of genetic content of GF-20 with some relatives based on metabolic pathways.

| Metabolisms | Genes | *Ferriphaselus amnicola* | | *Ferriphaselus sp.* | | *Gallionella capsiferriformans* | *Sideroxydans lithotrophicus* |
| --- | --- | --- | --- | --- | --- | --- | --- |
|  |  | F-20 | OYT1 | IN19 | CF-38 | ES-2 | ES-1 |
| Iron oxidation | *mtoAB* | nd | - | nd | nd | + | + |
|  | *mtrD* | nd | - | nd | nd | + | - |
|  | *pioAB* | nd | - | nd | nd | - | - |
|  | *cymA* | nd | - | nd | nd | - | + |
|  | *cyc1* | nd | - | nd | nd | - | - |
|  | *cyc2* | + | + | + | nd | - | - |
|  | *actAB* | + | + | + | + | + | + |
| Sulphur oxidation | *soxAXYZB* | nd | - | nd | nd | - | + |
|  | *dsrAB* | + | + | + | nd | - | + |
|  | *dsrHFE* | + | - | + | nd | - | + |
|  | *sat* | nd | - | nd | nd | - | + |
|  | *aprAB* | nd | - | nd | nd | - | + |
|  | *soeABC* | nd | - | nd | nd | - | - |
|  | *cysNDCHJI* | + | + | + | + | + | + |
|  | *ttr* | nd | + | nd | nd | - | - |
|  | *ssu* | nd | + | nd | nd | + | - |
|  | *sbp* | nd | + | nd | nd | - | - |
|  | *sqr* | + | + | nd | nd | - | - |
| CO_2_ fixation | RuBisCO (Form II) | + | + | + | + | + | + |
| Nitrogen fixation | *nifDEKNXHT* | + | + | + | + | - | + |
| Hemerythrins | trHbs | 2 | 4 | 2 | 2 | 3 | 14 |
| Motility & Chemotaxis | GGDEF domain | 16 | 9 | 49 | 24 | 46 | 43 |
|  | Histidine kinase | 17 | 8 | 10 | 6 | 47 | 37 |
|  | *luxR* | + | + | nd | nd | + | + |
|  | *cheAWZY* | + | + | + | + | + | + |
|  | *aer* | + | + | nd | nd | + | + |
|  | *fliGMN* | + | + | + | + | + | + |
|  | *motABC* | + | + | + | + | + | + |
|  | Pilin genes | + | + | + | + | + | + |
| Oxygen defense | *sodB* | + | + | + | + | + | + |
|  | *Cat* | nd | - | nd | + | - | + |
|  | peroxydase | 9 | 4 | 7 | nd | 3 | 1 |
| Metal sensitivity | *merATPF* | + | - | + | nd | + | - |
|  | *arsRCDA* | + | + | + | + | + | - |
|  | *czcA* | 6 | 6 | 4 | 5 | 8 | 2 |
|  | Cations pumps | 2 | 0 | 0 | 0 | 5 | 2 |
| Polysaccharide synthesis | Glycosyl transferase | 13 | 4 | 20 | 19 | 8 | 9 |
|  | Epimerase/deshydratase | 9 | 9 | 5 | 4 | 3 | 6 |
|  | *xagBCD* | + | + | + | + | - | - |
|  | *bcsBZC* | + | + | + | + | + | - |
|  | *algJF* | + | - | nd | nd | + | + |
| Phage and recombination | integrase | 22 | 11 | 18 | 9 | 21 | 3 |
|  | transposase | 14 | 15 | 16 | 20 | 25 | 3 |
|  | Mu-like prophage | nd | 2 | nd | nd | - | - |
|  | *traUGHFCB* | + | - | nd | nd | + | - |
| nd : incomplete genome |  |  |  |  |  |  |  |

**Table_S3_Supplementary_Data**. Percentage of fatty acids in the membrane composition of *Ferriphaselus amnicola* GF-20.

| Name | Nomenclature | Major ions | Composition (%) |
| --- | --- | --- | --- |
| Decanoic acid | C10:0 | **74**, 87, 143 | 0,48 |
| Dodecanoic acid | C12:0 | **74**, 87, 143, 171, 183 | 1,31 |
| Tetradecanoic acid | C14:0 | **74,** 87, 143, 199, 211, 242 | 3,44 |
| Pentadecenoic acid | C15:1 | **55**, 74, 87, 143, 199 | 0,37 |
| Pentadecanoic acid | C15:0 | **74**, 87, 143, 199, 213 | 3,18 |
| Hexadecenoic acid | C16:1 | **55**, 74, 96, 152, 194, 236 | 0,43 |
| Hexadecanoic acid | C16:0 | **74**, 87, 143, 227, 270 | 42,94 |
| Heptadecanoic acid | C17:0 | **74**, 87, 143, 199, 241, 284 | 2,00 |
| Octadecenoic acids | C18:3n3 C18:2n6t C18:1n9c | **55**, 69, 83, 180 | 0,90 |
| Octadecanoic acid | C18:0 | **74**, 87, 143, 199, 255, 298 | 24,42 |
| Eicosanoic acid | C20:0 | **74**, 87, 143, 283, 326 | 2,32 |
| Docosenoic acid | C22:1 | **55**, 69, 97, 207 | 0,46 |
| Docosanoic acid | C22:0 | **74**, 87, 143, 311, 354 | 2,32 |
| Tricosanoic acid | C23:0 | **74**, 87, 143, 207, 368 | 3,14 |
| Tetracosanoic acid | C24:0 | **74**, 87, 143, 207, 382 | 6,34 |
| Hexacosanoic acid | C26:0 | **74**, 87, 143, 207, 410 | 5,94 |

In bold, the ion used for the integration on the spectrum

**Table_S4_Supplementary_Data**. Abiotic and biotic Fe oxidation rates and relative contribution as a function of oxygen concentration**.**

| Oxygen concentrations (µM) | **Fe oxidation** | | | | | | | | |
| --- | --- | --- | --- | --- | --- | --- | --- | --- | --- |
|  | Rates (µM.h^-1^) | | | | | | Relative contribution (%) | | |
|  | Chemical | | | Biological | | | Chemical | Biological | Errors |
| 0 | 0.29 | \| ± \| \| --- \| | 0.16 | 0.35 | \| ± \| \| --- \| | 0.11 | 100.00 | 0.00 | 0.00 |
| 1 | 0.36 | ± | 0.24 | 10.29 | ± | 1.17 | 0.00 | 100.00 | 12.86 |
| 3 | 0.57 | ± | 0.13 | 12.96 | \| ± \| \| --- \| | 3.57 | 2.79 | 97.21 | 5.61 |
| 13 | 2.30 | ± | 0.40 | 13.62 | ± | 0.96 | 20.58 | 79.42 | 6.56 |
| 26 | 1.51 | ± | 0.37 | 10.55 | ± | 0.77 | 21.99 | 78.01 | 5.70 |
| 44 | 2.78 | ± | 0.74 | 5.53 | ± | 0.41 | 38.45 | 61.55 | 4.04 |
| 58 | 4.19 | ± | 0.9 | 0.00 | ± | 0.00 | 100.00 | 0.00 | 0.00 |

**Table_S5_Supplementary_Data**. AAI. ANI and DDH values calculated from pairwise comparisons of genomes and MAGs of *Ferriphaselus amnicola.*

| **AAI** | *Ferriphaselus amnicola* GF-20 | *Ferriphaselus amnicola* OYT1^T^ | *Ferriphaselus amnicola* IN18 | *Ferriphaselus* CF-38 | *Ferriphaselus globulitus* R-1 |
| --- | --- | --- | --- | --- | --- |
| *Ferriphaselus amnicola* GF-20 | 100 |  |  |  |  |
| *Ferriphaselus amnicola* OYT1^T^ | 97.08 | 100 |  |  |  |
| *Ferriphaselus amnicola* IN18 | 99.92 | 97.10 | 100 |  |  |
| *Ferriphaselus* CF-38 | 90.09 | 89.56 | 89.99 | 100 |  |
| *Ferriphaselus globulitus* R-1 | 85.15 | 85.17 | 85.15 | 84.62 | 100 |
| **ANI** | *Ferriphaselus amnicola* GF-20 | *Ferriphaselus amnicola* OYT1^T^ | *Ferriphaselus amnicola* IN18 | *Ferriphaselus* CF-38 | *Ferriphaselus globulitus* R-1 |
| *Ferriphaselus amnicola* GF-20 | 100 |  |  |  |  |
| *Ferriphaselus amnicola* OYT1^T^ | 96.10 | 100 |  |  |  |
| *Ferriphaselus amnicola* IN18 | 99.91 | 96.19 | 100 |  |  |
| *Ferriphaselus* CF-38 | 87.98 | 87.41 | 88.11 | 100 |  |
| *Ferriphaselus globulitus* R-1 | 83.36 | 82.45 | 82.28 | 81.69 | 100 |
|  |  |  |  |  |  |
| **DDH** | *Ferriphaselus amnicola* GF-20 | *Ferriphaselus amnicola* OYT1^T^ | *Ferriphaselus amnicola* IN18 | *Ferriphaselus* CF-38 | *Ferriphaselus globulitus* R-1 |
| *Ferriphaselus amnicola* GF-20 | 100 |  |  |  |  |
| *Ferriphaselus amnicola* OYT1^T^ | 68.80 | 100 |  |  |  |
| *Ferriphaselus amnicola* IN18 | 98.50 | 67.30 | 100 |  |  |
| *Ferriphaselus* CF-38 | 32.30 | 31.30 | 30.40 | 100 |  |
| *Ferriphaselus globulitus* R-1 | 24.50 | 24.30 | 24.50 | 24.10 | 100 |


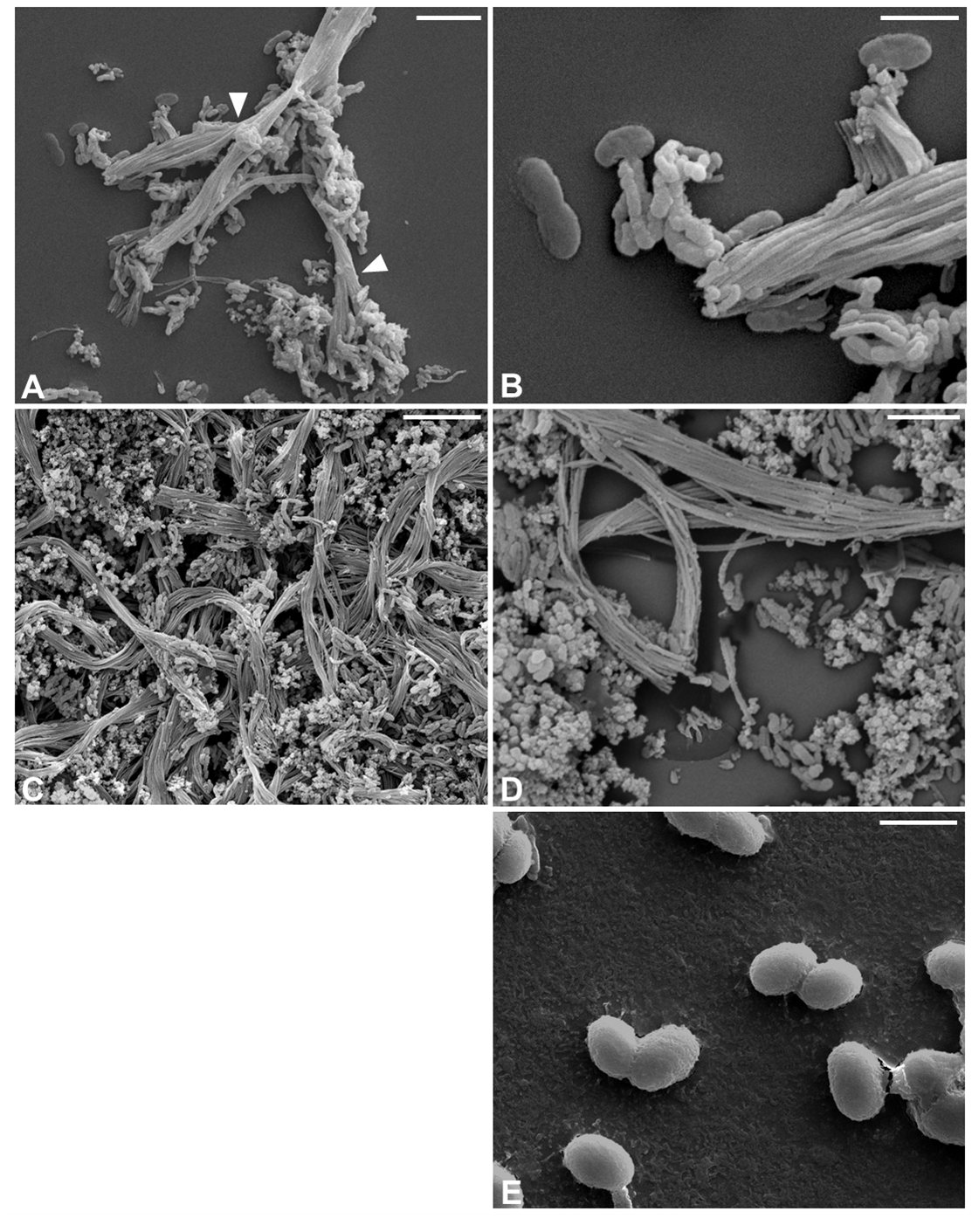


**Figure S1. SEM images of GF-20 cultures. (A and B)** Culture of GF-20 with only FeCl_2_. scale bar : 5 µm and 2.5 µm. respectively. **(C and D)** Culture of GF-20 with FeCl_2_ and thiosulfate. scale bar : 5 and 2.5 µm. respectively. **(E)** Culture of GF-20 with only thiosulfate. scale bar : 1 µm. White arrows indicate branching in the extracellular stalks that reflect cell division events.


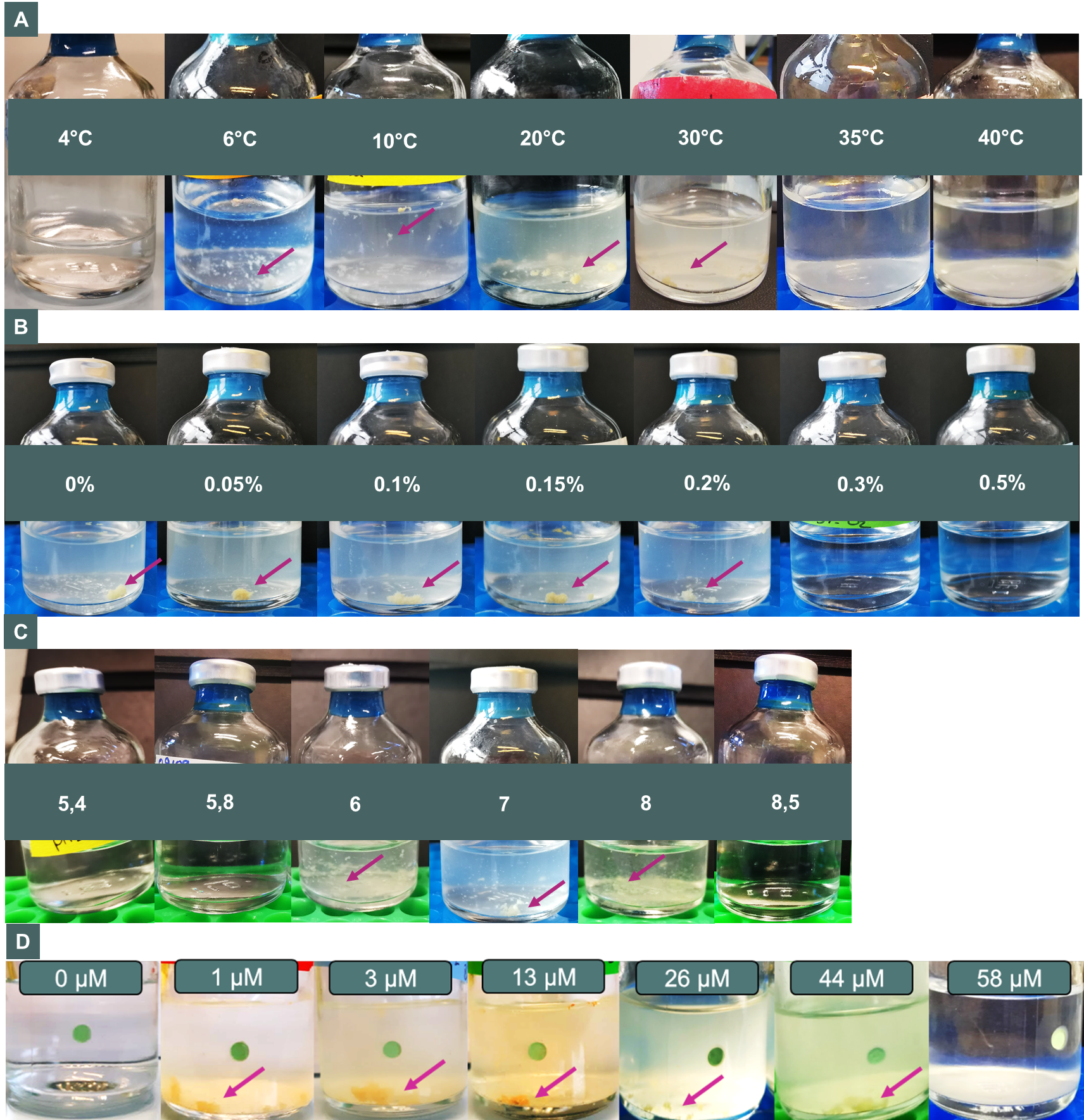


**Figure_S2_Supplementary_Data.** Pictures of GF-20 incubation experiments to determine ranges and optima of growth in function of **(A)** temperature. **(B)** salinity (NaCl concentration w/v)**(C)** pH and (**D**) O_2_. Growth of GF-20 was determined by the presence of microbial flocs (pink arrows) after 72 hours of incubation.

**
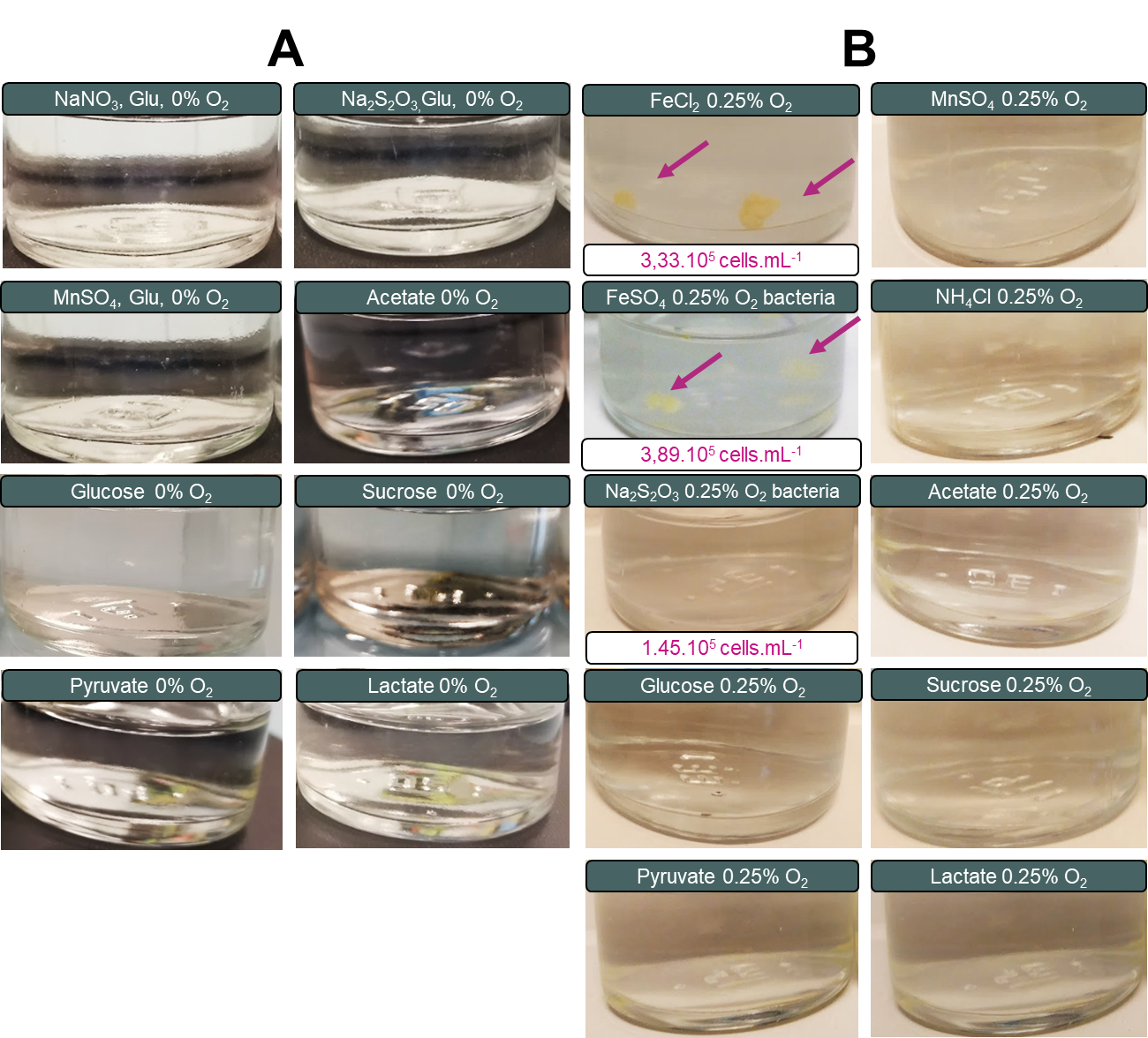
**

**Figure_S3_Supplementary_Data.** Pictures of GF-20 cultures in **(A)** anaerobic conditions with different electron acceptors and **(B)** suboxic conditions with different electron donors. Abbreviations: Glucose (Glu). Pictures were taken at the end of the incubation experiments. The final cell abundances were measured and values ​​are shown where growth occurred. Pink arrows indicate the presence of microbial flocs (incubation with FeCl_2_ or FeSO_4_).

**
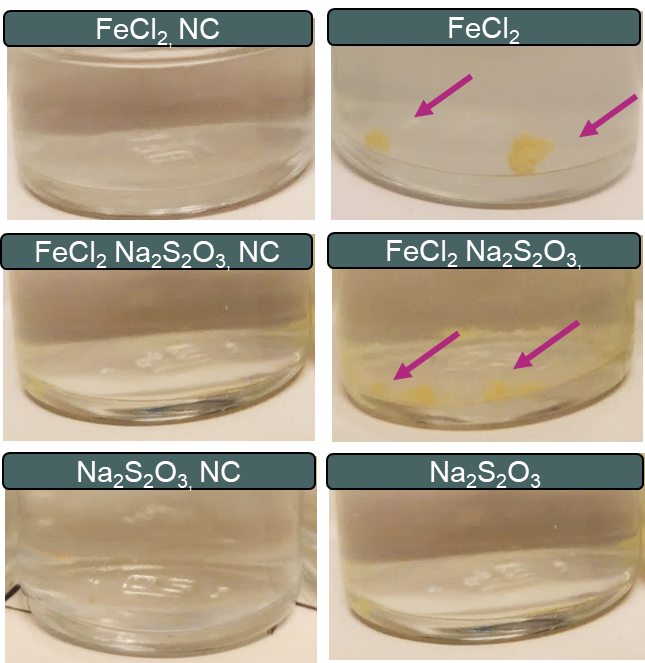
**

**Figure_S4_Supplementary_Data.** Pictures of GF-20 incubations with only FeCl_2_. with FeCl_2_ and thiosulfate and with only thiosulfate in suboxic condition. Pictures were taken at the end of the incubation experiments. NC : Negative controls without bacteria. Pink arrows show the development of microbial flocs.

**
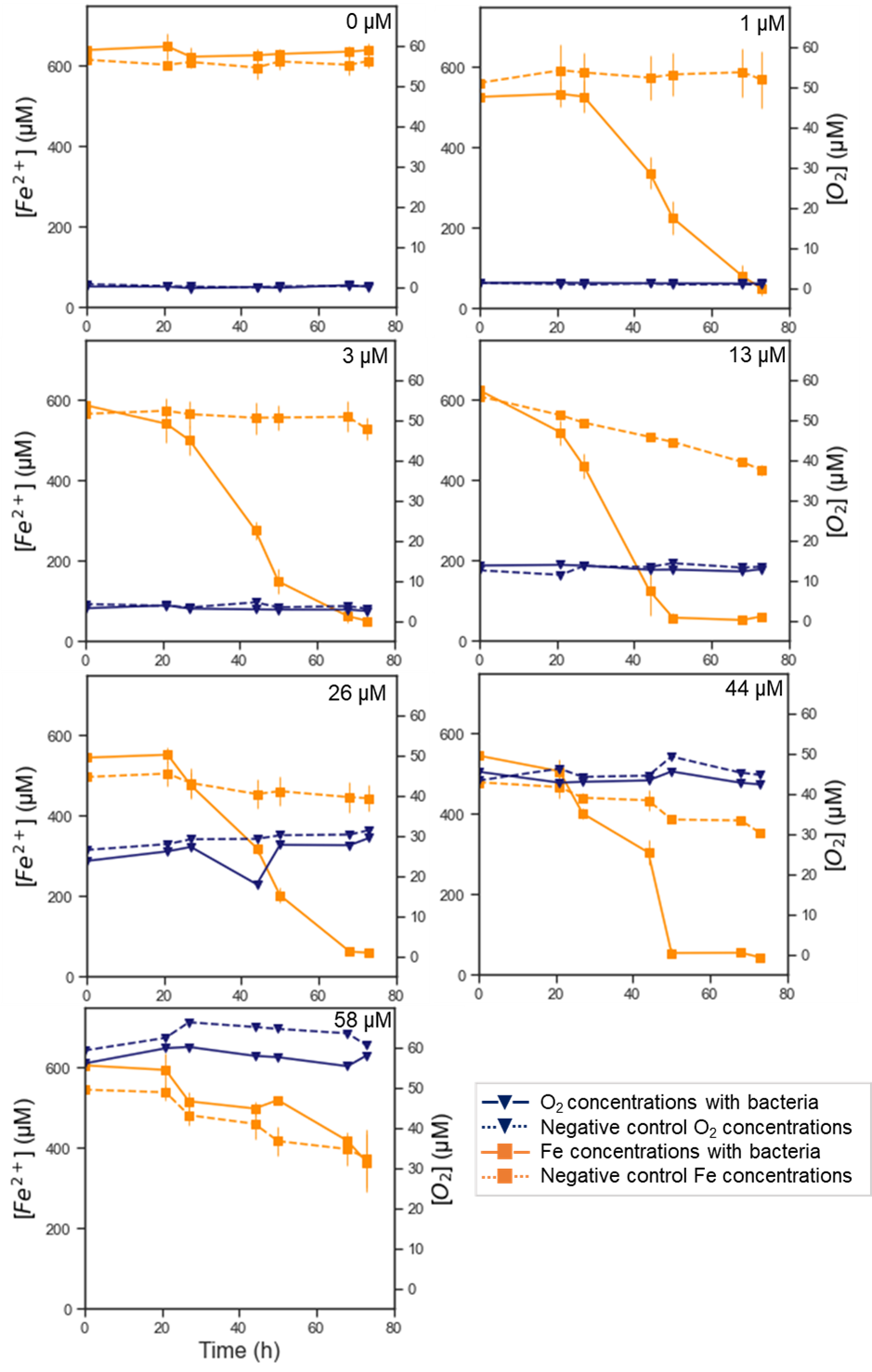
Figure_S5_Supplementary_Data.** Variation of Fe(II) concentration in GF-20 cultures incubated with seven different oxygen concentrations. Values represented in the plots are the averages of the measurements made on the triplicates. Error bars correspond to the standard deviations.

**REFERENCES**

Maisch M. Lueder U. Laufer K *et al.* Contribution of Microaerophilic Iron(II)-Oxidizers to Iron(III) Mineral Formation. *Environ Sci Technol* 2019;**53**:8197–204.

Tamura H. Goto K. Nagayama M. The effect of ferric hydroxide on the oxygenation of ferrous ions in neutral solutions. *Corrosion Science* 1976;**16**:197–207.
